# Water-cooled magnetic coupling drive attenuator design for wiggler light source in the TPS 31A beamline

**DOI:** 10.1107/S1600577522002338

**Published:** 2022-04-01

**Authors:** Ming-Ying Hsu, Chien-Yu Lee, Hok-Sum Fung, Bo-Yi Chen, Gung-Chian Yin

**Affiliations:** a National Synchrotron Radiation Research Center, 101 Hsin-Ann Road, Hsinchu Science Park, Hsinchu 30076, Taiwan

**Keywords:** attenuator, projection X-ray microscope, magnetic coupling, water cooling

## Abstract

An attenuator is generally used to decrease the power of an X-ray beam and prevent damage to detector sensors and other optical components. Therefore, attenuators are designed using foil or gas to absorb light source power. In this project, a large aperture and a water-cooling attenuator system are construed for the TPS 31A Projection X-ray Microscope and Transmission X-ray Microscope beamline. The source size of the wiggler is 300 µm × 7 µm on TPS 31A. The X-ray beam size at the sample position is 50 mm × 20 mm, located 49.5 m from the source. The light emission power is 1000 W in white-beam operation mode. The attenuator is needed to absorb energy for the light source and it has 12 foil carriers. The absorption foil size is 56 mm × 46 mm for the beam size across different beamline operation modes, and the cooling capacity is greater than 1000 W. This study applies a magnetic coupling-type attenuator system with foil carrier cooling carried out by the side chamber walls without the feedthrough having water enter the chamber to solve the thermal dissipation issue.

## Abstract 2

A water-cooled magnetic coupling attenuator design is presented.

## 1. Introduction

**Figure 1:**
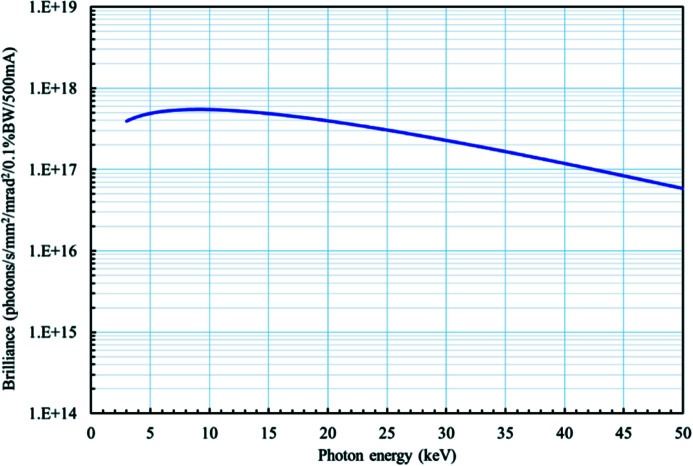
Brilliance of the TPS 31A wiggler, W100.

The Taiwan Photon Source (TPS) is one of the world’s brightest synchrotron X-ray sources. The brilliance and photon flux of the TPS 31A insert device are shown in Fig. 1 ▸. The TPS storage ring comprises 24 double-bend achromat (DBA) cells with six straight sections of 12 m length and 18 straight sections of 7 m length. In TPS phase II, there are plans to build ten new beamlines. Among these are the TPS 31A Projection X-ray Microscope (PXM) and the Transmission X-ray Microscope (TXM) beamlines. Construction of the PXM beamline station finished in December 2021.

**Figure 2:**
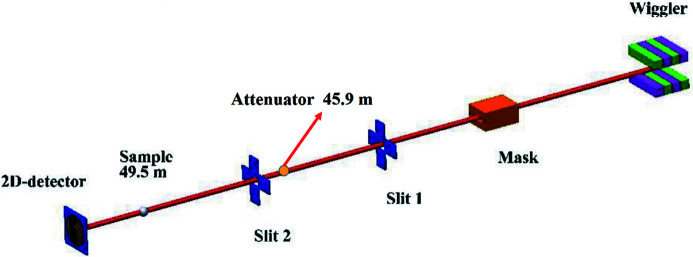
PXM in white-beam mode: the mask is at 24.3 m, the white-beam slit-1 is at 26 m, and the white-beam slit-2 is at 46.5 m.

Both PXM and TXM will feature W100 wigglers because their flux is approximately ten times higher than that of the bending magnet at a photon energy of 50 keV; the W100 parameters are shown in Table 1 ▸. The PXM beamline is designed to have an energy range of 5 to 50 keV. After the insert device, the white-beam mode power can reach 1000 W with an X-ray beam size at the sample position of 50 mm × 20 mm, located at 49.5 m, and with the attenuator 45.9 m before the white-beam slit. The PXM white-beam mode is shown in Fig. 2 ▸: the white-beam mask is at 24.3 m, the white-beam slit-1 is at 26 m, the white-beam slit-2 is at 46.5 m. The attenuator absorbs the lower X-ray beam energy or adjusts the beam magnitude to avoid damaging the PXM sample and sensor.

**Figure 3:**
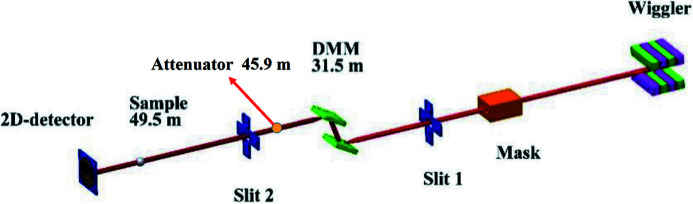
PXM in DMM mode.

**Figure 5:**
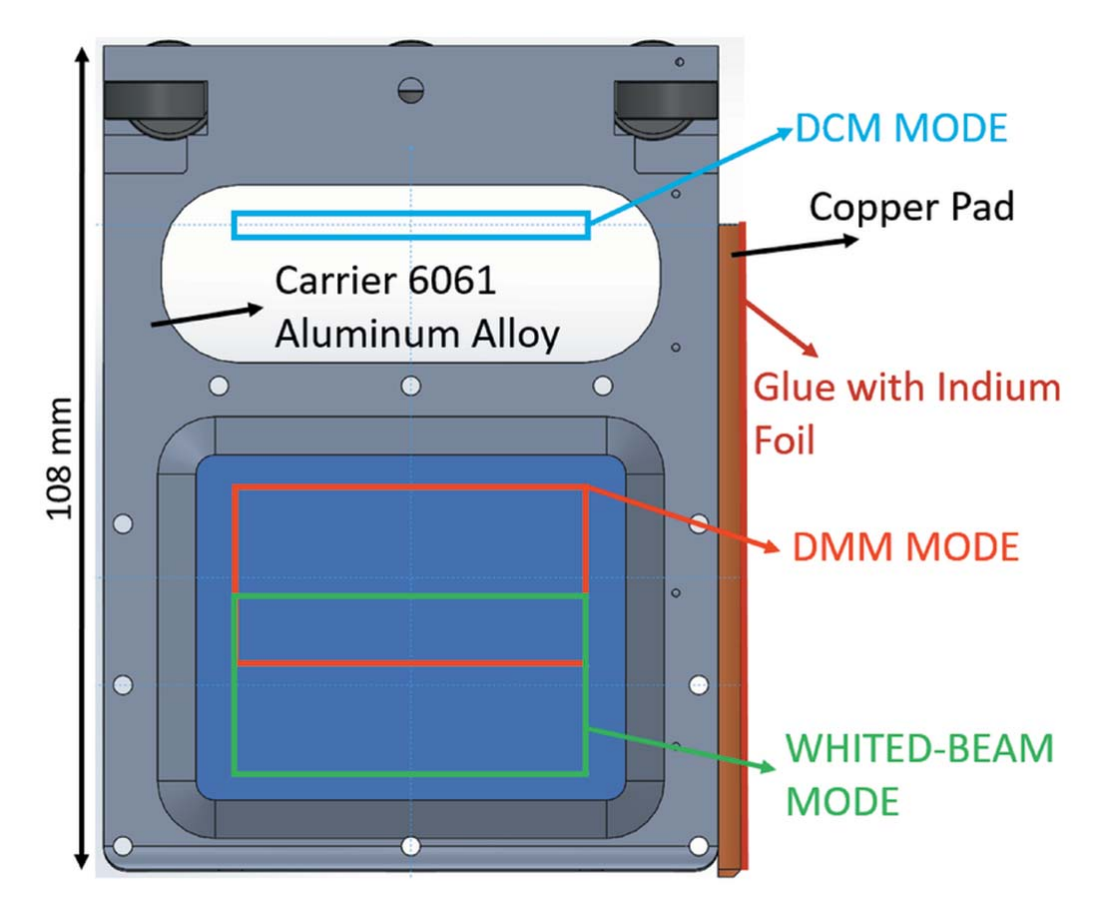
Diagram of the foil carrier and beam footprint of the foil carrier in different operation modes: double-crystal monochromator (DCM) mode, double-multilayer monochromator (DMM) mode, and white-beam mode.

With current commercial attenuator products such as JJ X-Ray, IRELEC, and ADC Inc., it is difficult to meet the requirements of both high absorbing power and large beam size. A double-crystal monochromator (DCM) or a double-multilayer monochromator (DMM) changes the beam’s vertical position. The foil window size of the attenuator is designed to be 56 mm (H) × 46 mm (V), allowing different operation modes of light through the attenuator. Figs. 3 ▸ and 4 ▸ show the DMM and DCM operation modes. The foil carrier design is shown in Fig. 5 ▸.

There are generally two types of design for the attenuator:

(*a*) The first uses an actuator, and a bellows moves the foil carriers into the beam path. Examples are at ESRF (https://www.esrf.fr/UsersAndScience/Experiments/MX/About_our_beamlines/ID23-1/TechnicalOverview/Optic/attenuators) and JJ X-Ray (https://www.jjxray.dk/p/attenuator/). Each actuator can carry one or several foil carriers, and a cooling system is combined with the actuator.

(*b*) In the second type of design, each foil carrier is moved into the beam path using magnetic coupling, and every actuator drives one foil carrier. The actuators that move the magnets are located outside the vacuum chamber and are usually pneumatic. Examples are at DESY (Seeck *et al.*, 2015 ▸) and IRELEC (https://www.irelec-alcen.com/en/synchrotrons/X-ray-beam-attenuators#no-back).

In this project, magnetic coupling is used to move a foil carrier into the beam path. The attenuator foil absorbs the beam power, and its thermal load path is from the foil to the carrier and then to the chamber wall. Because of the magnetic coupling, the attenuator chamber material is chosen to be 6061 aluminium alloy cut from bulk 6061 aluminium alloy. The outside of the chamber has a mini-channel fin water-cooling plate (https://www.qats.com/Products/Liquid-Cooling/Cold-Plates/ATS-CP-1000), and the rectangular mini-channel width is approximately 240 µm (ATS, Inc.). The pressure drop at a flow rate of 3.7 L min^−1^ (1 gallon min^−1^) is 5.998 kPa, cooling water is flowing in the cooling plate, and the cooling plate is in contact with the chamber wall via a bolt.

A magnetic-coupling-type attenuator system’s weakness is thermal cooling for high-power beam absorption. A bellows system has the problems of a larger attenuator foil, bellows and cooling water tube and the larger travel range of the attenuator. Therefore, this study applies side-surface contact of the foil carrier with the chamber wall to transmit the thermal load from the beam power. The foil carrier’s side surface that has a copper pad on it has indium foil glued to it, as shown in Fig. 5 ▸, thus increasing the surface contact ratio between the contact pad and the chamber wall. The glue (EP30AN-1) thermal conductivity is 3.31 W m^−1 ^K^−1^ and has low outgassing certifications from NASA. The glue is currently being applied in the TPS soft X-ray white-beam chamber. The foil carrier moves to chamber wall surfaces by the magnetic coupling pneumatic actuator. The foil carrier moving inside the aluminium chamber detects its two end positions using magnetic proximity sensors to ensure that the foil carrier is in the correct position.

## 2. Experimental model

**Figure 6:**
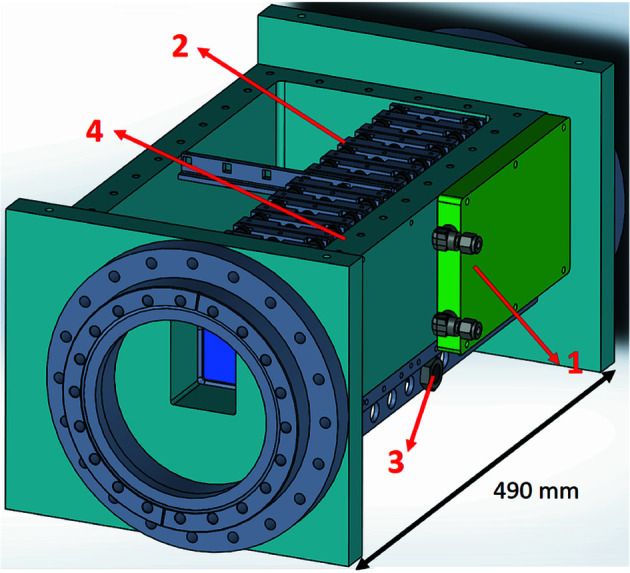
Diagram of the attenuator: (1) water cooling plate, (2) aluminium foil carrier, (3) pneumatic actuator, and (4) chamber cooling sidewall.

The design of the attenuator system is shown in Fig. 6 ▸. An experimental model is necessary to verify that the design is successful. The attenuator has 12 foil carriers and 12 ball-bearing slides and the system’s total length is 574 mm. The experimental model has a length of 71 mm with one foil carrier and one slide.

**Figure 7:**
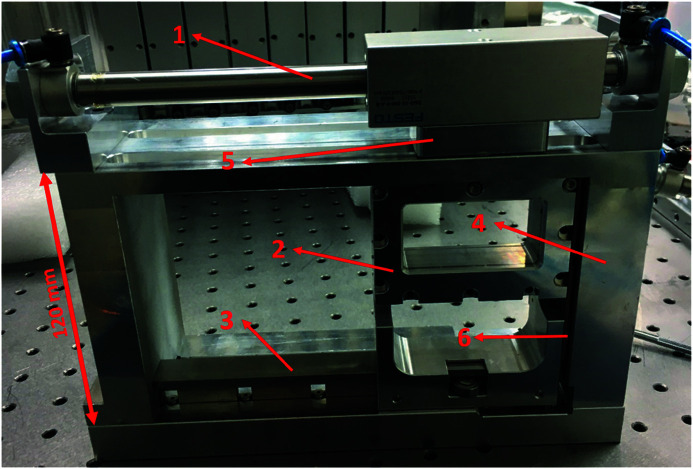
Experimental model of the attenuator: (1) pneumatic actuator, (2) aluminium foil carrier, (3) ball-bearing slide, (4) chamber cooling sidewall, (5) magnet set, and (6) copper pad.

The actual system is shown in Fig. 7 ▸. The ball-bearing slide is assembled with six ball bearings, and each ball bearing (SE624ZZSTPRC3, KOYO) is lubricated with a fluoro­polymer coating on the entire surface. The system can achieve a vacuum pressure of 1 × 10^−7^ mbar. The ball bearings can be changed to a high vacuum type, and the attenuator system can reach a vacuum pressure of 1 × 10^−12^ mbar if the attenuator needs to be set near the beam exit port.

**Figure 8:**
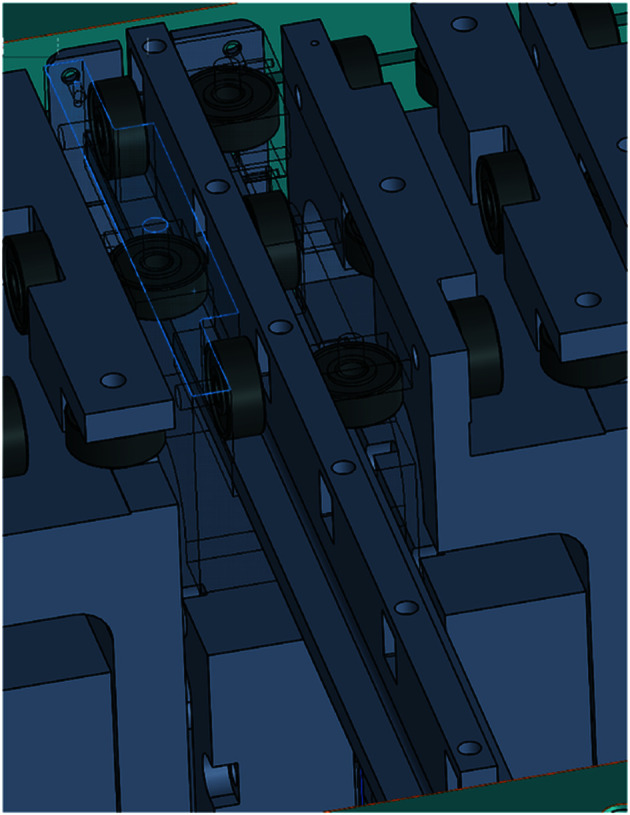
Diagram of the foil carrier and six-ball-bearing slider.

The pneumatic actuator (DGO-12-100-P-B, FESTO) slide travel range is 100 mm, and it moves the outside of the chamber and brings the magnet to move the foil carrier inside the vacuum chamber. The magnet set comprises two neodymium magnets (MGLN20-10-10, MISUMI). The magnet set and magnet are located outside the vacuum chamber, without direct contact with the vacuum chamber. The magnet still functioned after baking in the vacuum chamber at a temperature of 200°C in the commission test. Each magnet has size 20 mm × 10 mm × 10 mm, and the surface magnetic flux density is 4700 G. The gap inside the vacuum chamber is 0.3 mm, the chamber wall thickness is 3 mm in the magnet moving area, and the gap outside the vacuum chamber is 1 mm. The test results with the experimental model show that the pneumatic actuator and magnet set outside the chamber smoothly moved the foil carrier, which demonstrates that the six-ball-bearing slider design is successful. A diagram of the foil carrier and the six-ball-bearing slider is given in Fig. 8 ▸. The magnetic contact force on the vacuum chamber wall is tested using Fuji Prescale Film (4LW; 0.5 kgf mm^−2^ to 2 kgf mm^−2^). The vacuum chamber wall can achieve an average contact area force of 70 kPa, the contact area ratio is greater than 80%, and the contact area is 1847 mm^2^.

**Figure 9:**
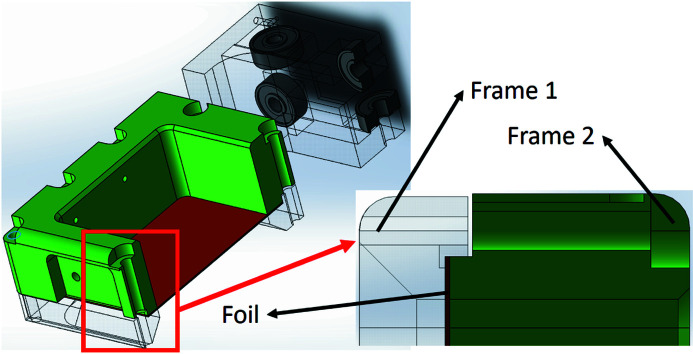
Diagram of the frame and foil clamp.

**Figure 10:**
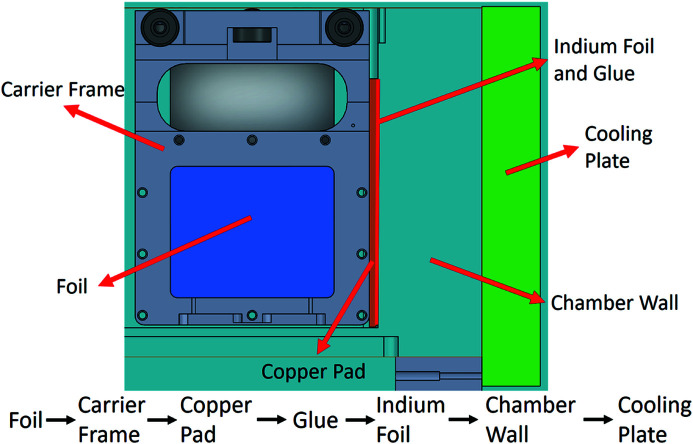
The thermal transfer path from the foil to the chamber wall.

The thermal design of the foil carriers is based on larger-size absorption foil and foil carriers. The foil is clamped between two frames, and the two frames are fixed with nine M3 bolts. The foil and frame contact area is 443 mm^2^ and the frame’s contact pressure force on the foil is 1 MPa over the contact area. The frame and foil clamp material is 6061 aluminium alloy, and the design is shown in Fig. 9 ▸. The absorption foil’s increased size can help decrease the maximum temperature in the absorption foil and also increases the foil carrier’s contact area with the absorption foil surface. A copper contact pad was designed for the foil carrier to contact the cooling wall. The contact pad area was 87 mm × 23 mm, and this large contact area can improve the heat transfer to the cooling wall. The thermal transfer path is from the film to the carrier frame, to the copper pad, to the glue, to the indium foil, to the vacuum chamber wall, and then to the water-cooling plate. The thermal path is shown in Fig. 10 ▸.

## 3. Thermal analysis

The attenuator absorbs heat from the light source. The wiggler source energy is 5 to 30 keV, and each foil’s estimated absorbed heat load is 100 W. The heat absorption from X-rays of the foil material can be calculated by (Thompson *et al.*, 2009 ▸)

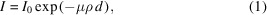

and 

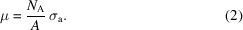

Equation (1) calculates the ray absorption energy after the foil, where *I*_0_ is the incident ray energy, *I* is the emergent ray energy after the foil absorption, μ is the mass absorption coefficient, ρ is the material density, and *d* is the foil thickness. The absorption coefficient for pure material is described by equation (2), where *N*_A_ is Avogadro’s number, *A* is the atomic weight, and σ_a_ is the total absorption cross-section. The absorbing foils are pure material, and equations (1) and (2) can help to calculate each foil’s thickness and heat load.

**Figure 11:**
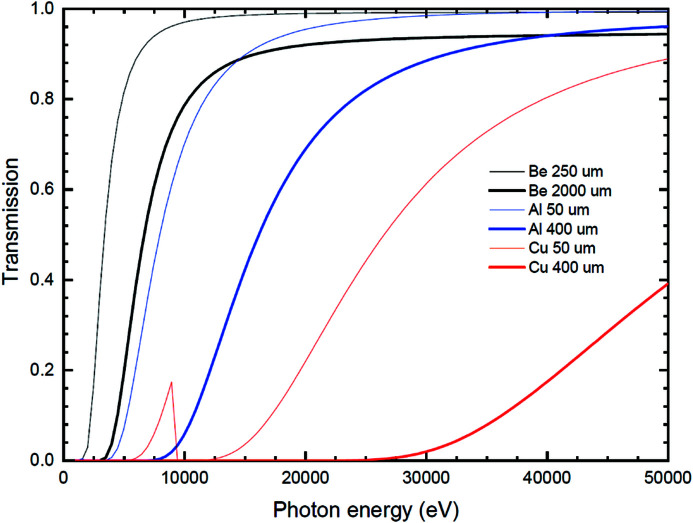
The different material and thickness absorption foils transmittance.

The thickness and material of each absorption foil are listed in Table 2 ▸. The attenuator system has 12 foil carriers and the foil order is decided by its material and thickness. Each foil absorbs approximately 100 W in this design. The atomic number of the small metal material is in front of the ranking of the foil carriers. The transmittance for different thicknesses of beryllium, aluminium and copper foil are shown in Fig. 11 ▸. The beryllium foil can filter out the low-energy flux below about 2.5 to 5 keV, the aluminium foil filter range below about 5 to 10 keV, and the copper foil filter range about 17.5 to 35 keV. The user can choose their experiment’s necessary energy range to change the absorption foil type. In the operation of the attenuator system, the aluminium foils are only allowed to be in the white beam after all the beryllium foils are in the white beam. The power absorbed by the material will melt the heavy metal foil in the white beam if there is no light metal foil in front of it.

The foil carrier absorbs the light source energy, and the heat load needs to be transferred to the chamber cooling wall. The primary way that heat is transferred in the vacuum chamber is thermal conduction (Lienhard & Lienhard, 2019 ▸), but the complete thermal conduction equation is complex,

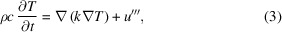

where ρ is the material density, *c* is the material heat capacity, *T* is the temperature, *k* is the thermal conductivity, and *u*′′′ is the internal heat generation. In the heat transfer process, the thermal conductivity *k* is constant and the simulation is in a steady state ( 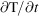 = 0), so equation (3) can be simplified to

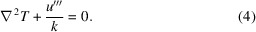

Equation (4) can be used to solve the thermal conduction problem, but the attenuator chamber, absorption foil and foil carrier geometry have many points of contact. Thus, finite-element method (FEM) software is applied to find the attenuator’s thermal distribution.

The different material contact forces and heat transfer coefficients are an important issue in this study, and several articles have been written about this (Grujicic *et al.*, 2005 ▸; Yovanovich, 2006 ▸; Milanez *et al.*, 2003 ▸). The thermal contact conductance is calculated by

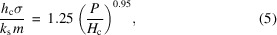




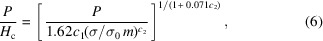




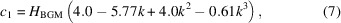




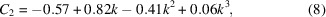




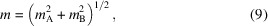




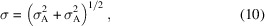




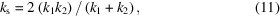

where *H*_BGN_ = 3.178 GPa is the geometric mean of the minimum and maximum values of *H*_B_ for the test materials, *k* = *H*_B_/*H*_BGM_, *H*_B_ is the Brinell hardness of the bulk material, *P* is the contact pressure (Pa), *m* is the combined mean absolute slope (radians), *m*_A_ and *m*_B_ are the material slopes (radians), *H*_c_ is the micro-hardness (Pa), *C*_1_ and *C*_2_ are the Vickers microhardness correlation coefficients, σ is the combined RMS roughness (m), σ_A_ and σ_B_ are the material RMS roughness values (m), *k*_s_ is the harmonic mean thermal conductivity (W m^−1^ K^−1^), *k*_1_ and *k*_2_ are the material thermal conductivities, and *h*_c_ is the contact conductance (W m^−2^ K^−1^).

The cooling side of the attenuator chamber can take a heat load of 1000 W with a temperature difference of 5.5°C between the inlet and outlet water. The simulation’s cooling wall temperature is set at 25°C because the cooling plate’s inlet water temperature is set at 17.5°C, the outlet water is 23°C, and a temperature difference of 2°C is assumed between the outlet water and the cooling pad surface so that the cooling capacity can reach 1000 W. The absorption foil material is beryllium, and the foil thickness is 0.4 mm. The heat load absorption of the foil is 100 W.

The foil carrier material is aluminium, and the side of the foil carrier has a copper contact pad on its surface. The copper contact pad surface is glued with 0.2 mm indium foil to increase the surface contact with the vacuum chamber’s cooling surface. The heat transfer between the vacuum chamber wall and copper pad surface is calculated using equations (5)–(11). The contact conductance between the absorption foil and foil carrier surface is *h*_foil&carrier_ = 68500 W m^−2^ K^−1^. The contact conductance between the carrier and copper pad surface is *h*_carrier&pad_ = 21972 W m^−2^ K^−1^. The contact conductance between the indium foil and vacuum chamber wall surface is *h*_In&wall_ = 1570 W m^−2^ K^−1^. The parameters used to compute the micro-contact thermal resistance are shown in Table 3 ▸.

## 4. Simulation results

**Figure 12:**
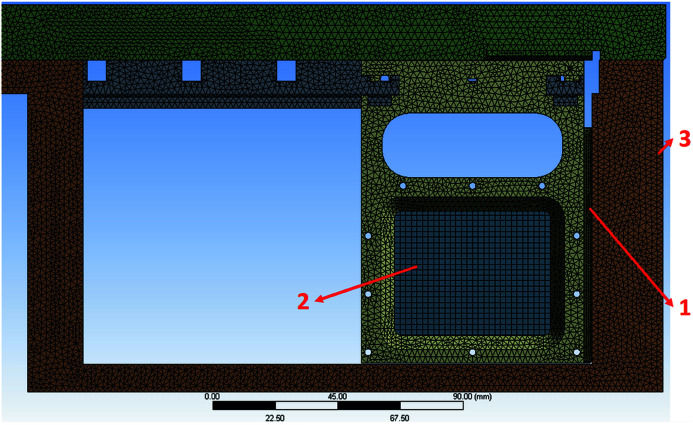
Simulation model and grid: (1) copper contact pad, (2) absorption foil (100 W), and (3) chamber cooling side (25°C). The total number of grid points is 1115215.

The simulation was performed using an experimental model to calculate the thermal distribution. The full attenuator system model needs more than ten times the number of grids as the experimental model. The grid size also increases the computer memory size requirements and the calculation time. The boundary conditions are a heat load absorption of the foil of 100 W. The thickness of the glue between the indium foil and copper pad is 20 µm and the thermal conductivity is 3.31 W m^−1^ K^−1^. The foil carrier contact area is 443 mm^2^. The water-cooling plate is supplied using a water chiller and it is easy to maintain an external wall temperature of 25°C for the vacuum chamber. The number of grid points in the FEM simulation is 1115215 and Fig. 12 ▸ shows the grid’s distribution.

**Figure 13:**
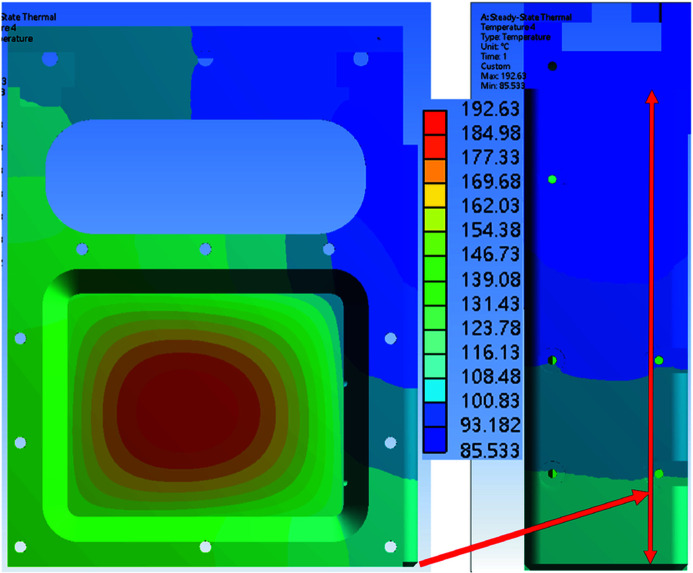
Numerical simulation results for temperature distribution of the foil carrier and copper contact pad with a contact area of 60%.

**Figure 16:**
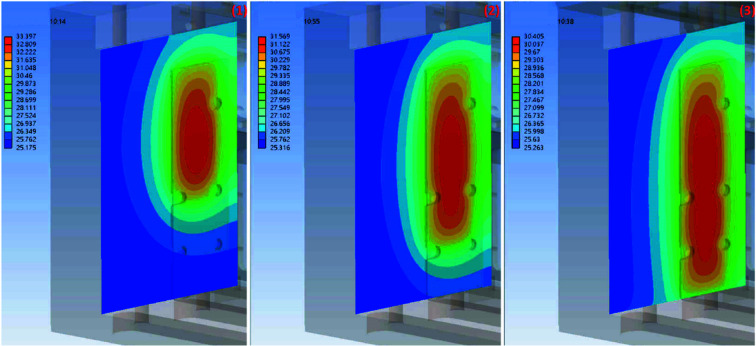
Numerical simulation results with for temperature distribution of the chamber wall contact area, (1) contact ratio 60%, (2) contact ratio 80% and (3) contact ratio 100%.

The simulation results were obtained with different copper contact area ratios of 60%, 80% and 100%. The copper contact pad and absorption foil are part of the foil carrier. Figs. 13 ▸, 14 ▸ and 15 ▸ show the simulation results for the temperature distribution in the foil carrier and contact pad. The foil carrier’s maximum temperature is in the absorption foil. The copper contact pad’s maximum temperature is the main heat transfer area, and a high contact ratio can reduce the temperature difference in the contact pad surface. The temperature difference between the contact pad and chamber wall is increased by the contact ratio decline. The contact area of the chamber wall temperature distributions is shown in Fig. 16 ▸.

The chamber walls and contact pad’s minimum and maximum temperatures are shown in Table 4 ▸. The temperature difference changes linearly with the contact ratio. The copper contact pad is designed to have a maximum temperature of less than 156.6°C (the melting point of indium) because the contact pad surface with the 0.2 mm indium foil increases the contact ratio with the cooling wall. The contact pad’s temperature with a 60% contact ratio is still lower than the design goal.

## 5. Conclusion

TPS 31A is one of the most potent projection X-ray microscope beamlines in the world. The high photon flux means that the problem of heat generation needs to be addressed. Therefore, this study improved upon a previous magnetic-coupling-type attenuator system that did not have a cooling system. The heat load from the beamline can be transferred from the absorption foil to the water-cooling pad outside of the vacuum chamber. Thus, the attenuator system can be small and compact, and the large absorption foil and foil carrier size can decrease the thermal load density and maximum system temperature.

**Figure 17:**
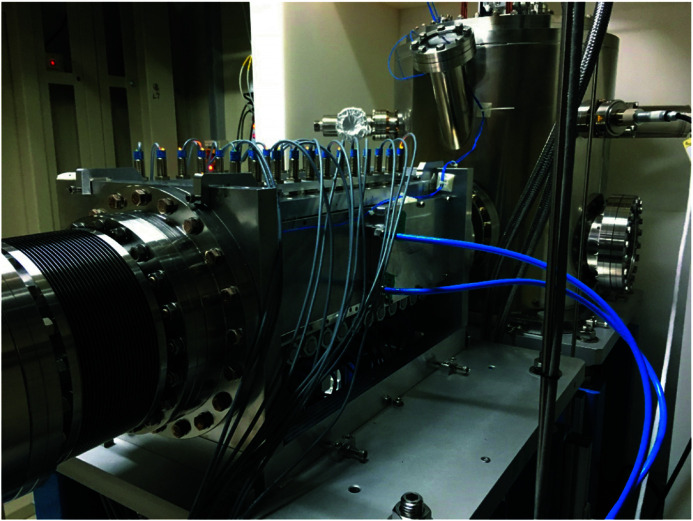
Attenuator system installed in the TPS 31A beamline.

Fig. 17 ▸ shows the attenuator system installed in the TPS 31A beamline. The contact pad surface had a 0.2 mm indium foil glued to it, which improved the cooling surface’s contact ratio. In the offline test of the attenuator system, the cooling surface contact ratio was higher than 80%. In the simulation result, when the contact pad contact ratio was 60%, the foil carrier’s maximum temperature was 192.63°C, which is below the system design goal of 200°C. The copper contact pad’s temperature with the 60% contact ratio was 110.74°C, which is lower than 156.6°C (melting point of indium). The maximum capacity of TPS 31A white beam mode is 1000 W, and the attenuator design meets the system requirement. For further user requests, the attenuator system can add several extra thermocouples to measure foil carrier temperature and an independent angle valve to maintain the system vacuum.

## Figures and Tables

**Figure 4:**
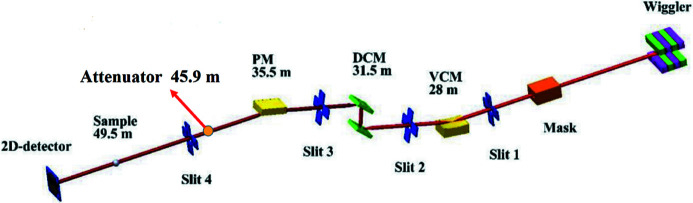
PXM in DCM mode.

**Figure 14:**
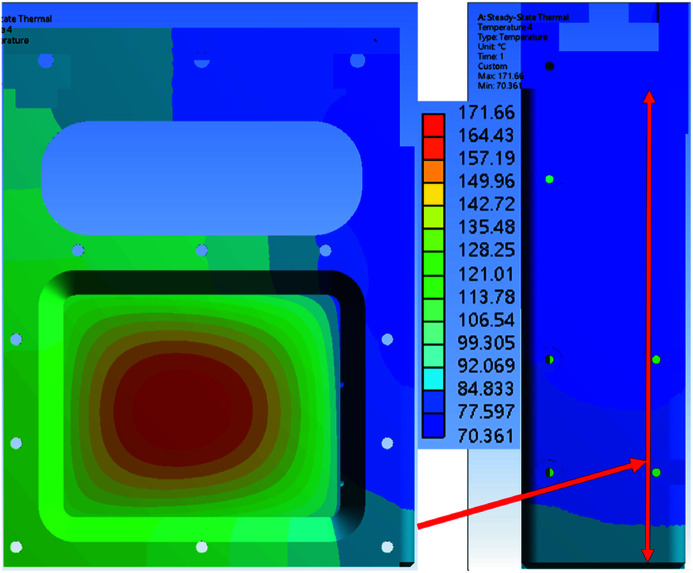
Numerical simulation results for temperature distribution of the foil carrier and copper contact pad with a contact area of 80%.

**Figure 15:**
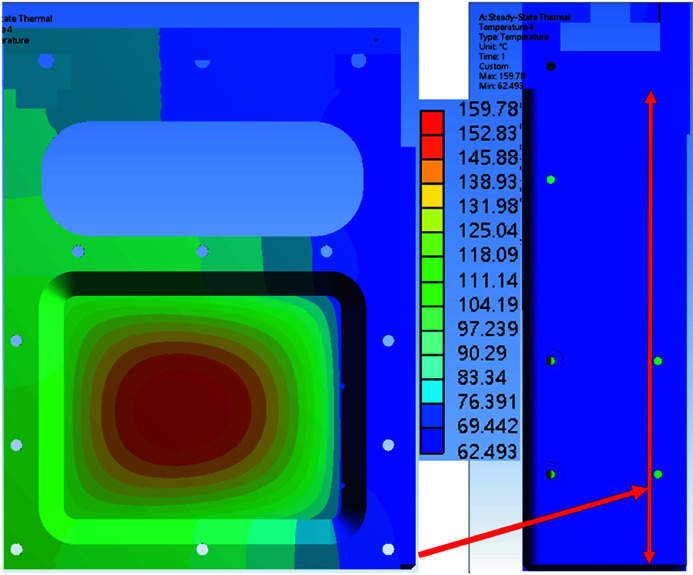
Numerical simulation results with for temperature distribution of the foil carrier and copper contact pad with a contact area of 100%.

**Table 1:** Parameters of the TPS insert device and wiggler

Wiggler	W100
Photon energy (keV)	5–50
Current (mA)	500
Peak field strength (T)	1.81
Period length (mm)	100
Number of periods (number of poles)	4 (8)
Total magnetic length, *L* (mm)	600
Pole gap (mm)	14
Deflection parameter, *k*	16.91
Total radiation power (kW)	3.7

**Table 2:** List of absorption foils

Foil number	Thickness (µm)	Material
1	250	Beryllium
2	500	Beryllium
3	1000	Beryllium
4	2000	Beryllium
5	50	Aluminium
6	100	Aluminium
7	200	Aluminium
8	400	Aluminium
9	50	Copper
10	100	Copper
11	200	Copper
12	400	Copper

**Table 3:** Parameters used to compute the micro-contact thermal resistance (Yovanovich, 2006 ▸)

Indium	
RMS surface roughness, *s* (m)	0.8 × 10^−6^
Brinell hardness, *H*_B_ (MPa)	10
Surface slope	0.1
Thermal conductivity of indium, *k* (W m^−1^ K^−1^)	86
External pressure to chamber wall, *F* (kPa)	70

Copper	
RMS surface roughness, *s* (m)	0.8 × 10^−6^
Brinell hardness, **H*B* (MPa)	932
Surface slope	0.1
Thermal conductivity of copper, *k* (W m^−1^ K^−1^)	399
External pressure to foil carrier, *F* (kPa)	1000

Aluminium alloy 6061	
RMS surface roughness, *s* (m)	0.8 × 10^−6^
Brinell hardness, *H*_B_ (MPa)	250
Surface slope	0.1
Thermal conductivity of 6061, *k* (W m^−1^ K^−1^)	167
External pressure to foil carrier, *F* (kPa)	10000

**Table 4:** Foil carrier, contact pad and chamber wall temperatures for different contact area ratios The 100% contact area of the copper pad to chamber wall is 1847 mm^2^.

	100%	80%	60%
Foil carrier maximum temperature	159.78°C	171.66°C	192.63°C
Contact pad maximum temperature	68.76°C	86.34°C	111.74°C
Contact pad minimum temperature	62.49°C	70.36°C	85.53°C
Chamber wall maximum temperature	30.41°C	31.57°C	33.40°C
Chamber wall minimum temperature	25.26°C	25.32°C	25.18°C
